# Supplementary figures and images for: Fast cycling culture of the annelid model Platynereis dumerilii
Source: PLoS One. 2023 Dec 21;18(12):e0295290. doi: 10.1371/journal.pone.0295290 (PMC10735030; doi:10.1371/journal.pone.0295290)

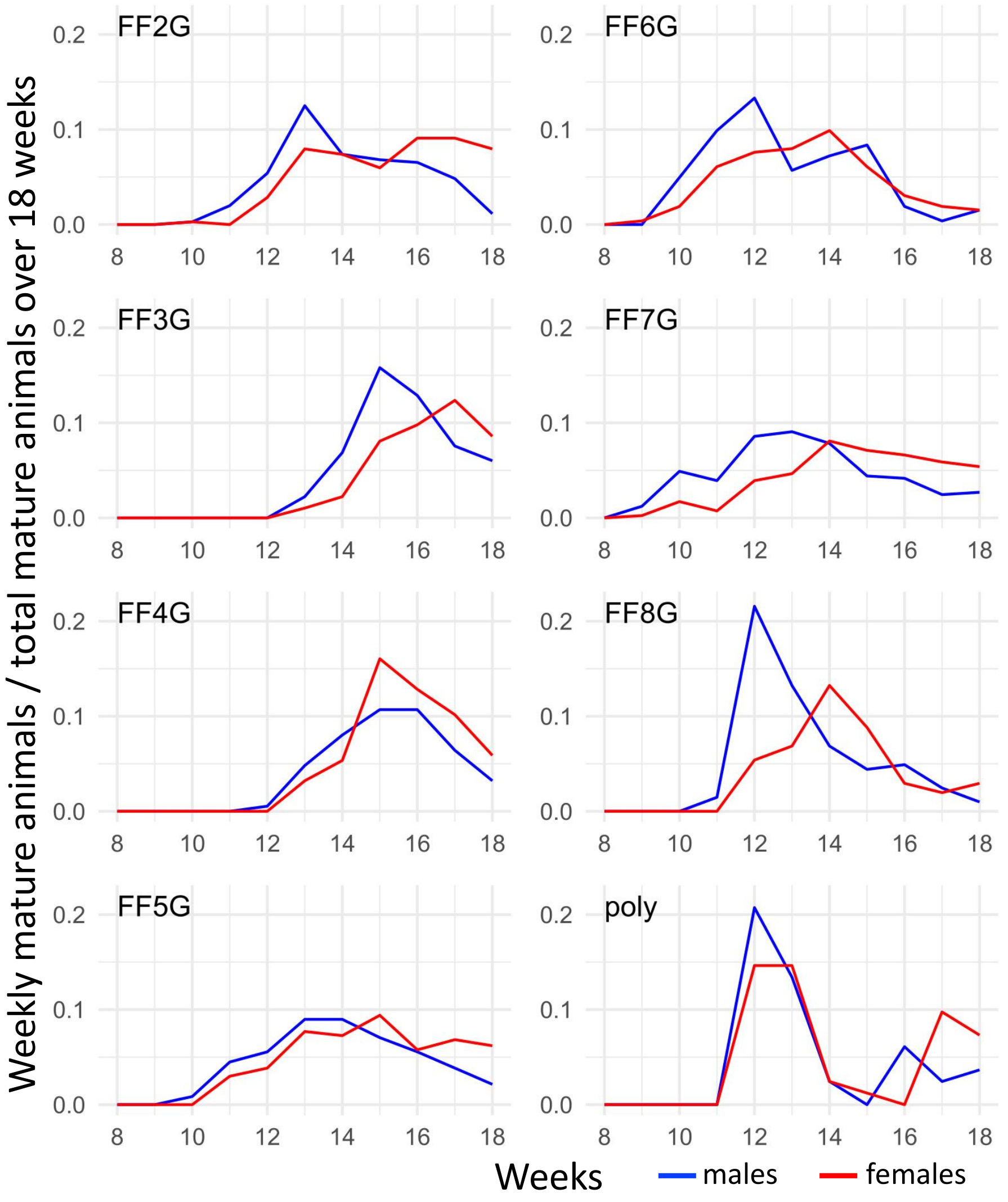

Supplement: S1 Fig — FF(2–8)G are the selected generation for which significant numbers of matures have been obtained. “poly” is the control culture with polymorphic worms raised with the same density and food regime as the last three selected FF generations. (TIF) [file pone.0295290.s001.tif]

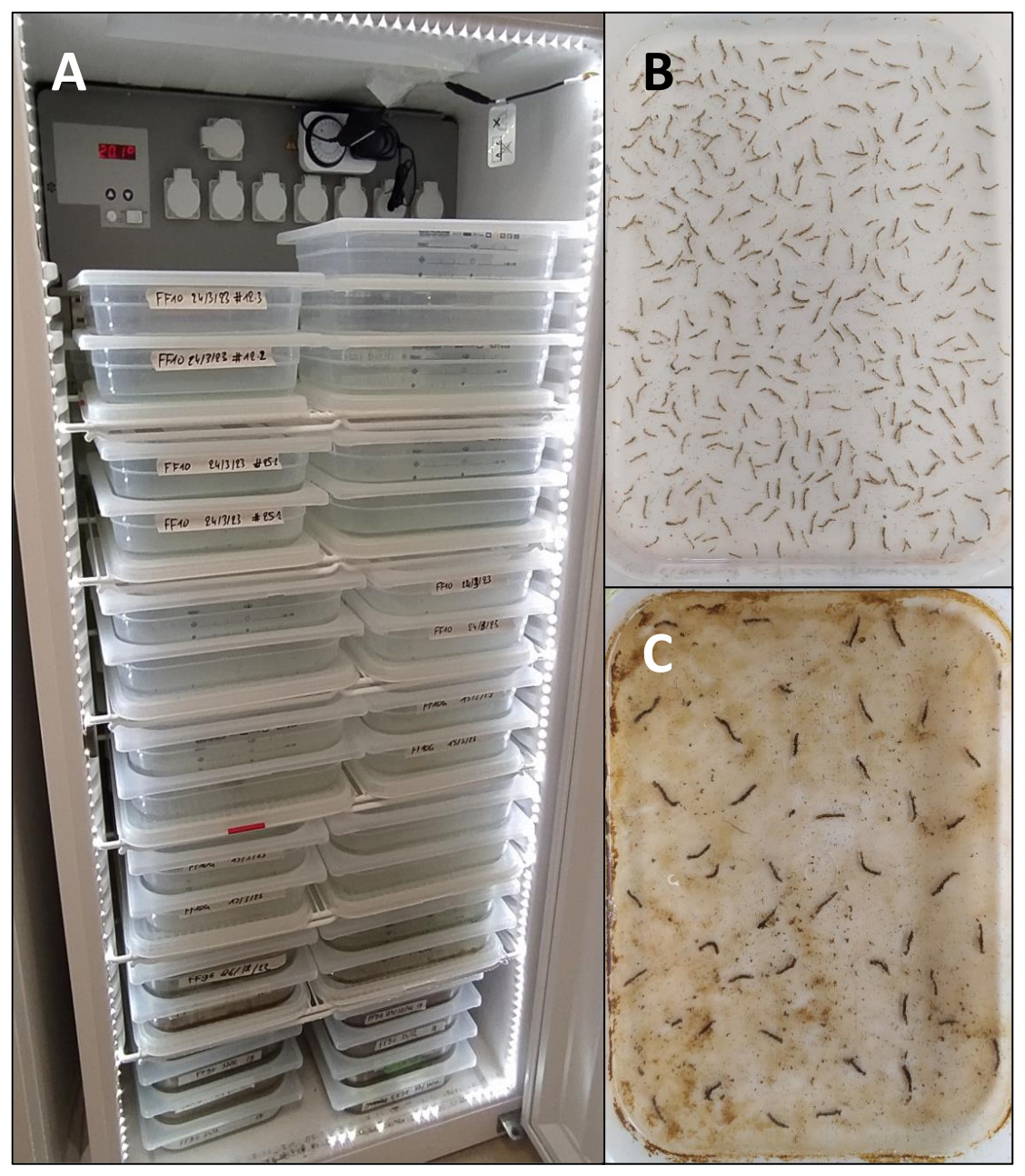

Supplement: S2 Fig — A. An open incubator is shown, displaying the stacking of worm boxes on shelves. Typically, boxes are stacked in pairs, allowing for the placement of 8 boxes on each shelf (half of the boxes are hidden in this photograph), totalling 50 boxes in the incubator. Take note of the LED ribbon dispensing warm white light, which is affixed all around the frame of the front door, approximately 2 cm from the edge, and connected to a mechanical timer (located at the top of the incubator). The box lids are secured in two corners, leaving the other two corners unfastened to facilitate gas exchange. A convenient method for feeding the boxes involves partially pulling out the shelves to access the inner boxes, and the unclipped corners are raised a few centimetres to allow the dispensing of food using a plastic pipette. B. This image shows a 2-month-old high-density box, illustrating the arrangement of the worm tubes, which are spaced apart from each other. C. In this photograph, a 2-month-old low-density box with 25 worms is depicted, highlighting the faster growth of the worms (notice the significantly longer tubes), which enables maturation to commence two weeks later. (TIF) [file pone.0295290.s002.tif]
