# Supplementary material for: Fast cycling culture of the annelid model Platynereis dumerilii
Source: PLoS One. 2023 Dec 21;18(12):e0295290. doi: 10.1371/journal.pone.0295290 (PMC10735030; doi:10.1371/journal.pone.0295290)
Supplement: S1 File — (DOCX) [file pone.0295290.s003.docx]

**Supplementary file 2 : troubleshooting guide**

| **Problem** | **Possible cause(s)** | **solution** |
| --- | --- | --- |
| Female release oocytes spontaneously; swimming female is “empty” | Manipulation stress can trigger oocyte release; released oocytes lose fertilization competence very quickly (< 1 min) and are therefore lost if not immediately fertilized. | Swimming female spotted in a box is kept in box until used for fertilization to avoid capture stress |
| Pair of worms doesn’t release gametes | One of the worms is not mature enough. | Replace with a fully mature actively swimming individual |
|  | One of the worms is exhausted after night-time swimming | Gently press the male with paintbrush to release the sperm and trigger the female |
|  | Pair is incompatible (genetic divergence ?) | Replace one of the worms with another swimming mature |
| All oocytes are unfertilized; the egg jelly is not released; no hexagonal pattern forms in the beaker | Pair is incompatible (genetic divergence ?) | None |
| Some oocytes are unfertilized; they lie in small groups beneath the fertilized egg jelly | Non optimal quality of sperm or eggs | After full release of egg jelly (1 hour), unfertilized oocytes can be removed by placing the beaker on an orbital shaker et moderate speed (80 rpm) for a few minutes. Oocytes will concentrate centripetally and can be pipetted out |
| At 48 hpf, some of the larvae do not develop normally; larvae swim in circles or spin (“spinners”); some larvae do not show the typical phototaxis characteristic of these stages | Non optimal quality of eggs / larvae | Abnormal larvae can be removed by placing the beaker on an orbital shaker et moderate speed (80 rpm) for a few minutes. Spinners will concentrate centripetally and can be pipetted out |
| Slow growth of juveniles; juveniles still tiny after two months | *Dimorphilus* contamination: dense population of tiny white worms at the bottom of the box | Extract *Platynereis* juveniles from box and rinse several times with NSW. Put the worms into a new box. Alternatively, kill the *Dimorphilus* in the contaminated box by exposing to deionized water for 5 mins. Replace with 500 ml of NSW and put back the *Platynereis* juveniles. |
|  | New box contaminated with industrial chemicals. | Do not use for human food!  New boxes are always left to soak in deionized water for at least 24 hrs and thoroughly brushed before first use. |
|  | Brownish bacterial film on water; water fouling | Change water. Skip feeding for one week. |
| Spontaneous reproduction in box, numerous tiny *Platynereis* growing in between sub-adults | Usually happens in weekends | No action required. Small worms do not grow fast enough to compromise the maturation of large ones. |
| Death of all worms, low level of water in box | Evaporation of water; sea water too salty | Lid has not been closed tight enough; ideally, it is clipped on one side and just laid on the other side but do not let a wide opening where air current can circulate. |
| Worms outside of tubes, inactive or curled | Probable water fouling from excess food | Change water. Skip feeding for one week. |
