# Supplementary material for: Fast cycling culture of the annelid model Platynereis dumerilii
Source: PLoS One. 2023 Dec 21;18(12):e0295290. doi: 10.1371/journal.pone.0295290 (PMC10735030; doi:10.1371/journal.pone.0295290)
Supplement: S2 File — (DOCX) [file pone.0295290.s004.docx]

**Supplementary file 2 : food, supply and equipment**

| **Equipment + material** | **reference number** | **provider** |
| --- | --- | --- |
| Thermostatic incubator | TC 445 S | Lovibond |
| LED strip warm white light | 320LED/M*5M | TTWAY |
| Polypropylen boxes GN 1/2 H. 65 mm | BACPOLYP12065 | Gastroland |
| Mechanical timer switch | 491004 | Expert Line |
| Plastic Pasteur pipettes 3 ml |  | Samco Scientific |
| Glass dish, 300 ml, D95mm, H55mm | 213114402 | Schott DURAN |
| Filtration unit, presterilized, 1 liter, 0.22 μm | S2GPU11RE | Millipore |
| Polypropylene tubes 50 ml | 431720 | Corning |
| 80-micron monofilament nylon mesh | SIIC-CNMO-80 | Filterworkshop.com |
| Natural 0.2 μm filtered sea water, 10 liter cans |  | Odemer |
| 100x antibiotic mix (10 000 U/ml penicillin; 10 000 µg/mL streptomycin) | 15140122 | Thermofisher |
| MgCl_2_ . 6 H_2_O | 1374248 | Sigma Aldrich |
| TetraMin flakes | T512425 | Tetra (Germany) |
| Sera micron |  | Sera (Germany) |
| Organic spinach, frozen, 50 g pellets, 600g package |  | Picard (France) |
| Tetraselmis frozen concentrate | TET 3600 | reedmariculture.com |

**Preparation of the food suspensions:**

Spinach (50g/l): A 50 g pellet is placed in a 1-liter beaker with 500 ml of NFSW to thaw. The mixture is then prepared using a hand blender and aliquoted into 50 ml polypropylene tubes. Spinach fragments tend to float, so it is necessary to mix thoroughly before aliquoting. The 50 ml tubes can be stored at -20°C until ready for use.

Tetramin (10g/l): This is a preparation of flakes that need to be ground. To achieve this, use a mortar and pestle. Grind thoroughly until you obtain a fine powder. Stocks of Tetramin powder are stored in 50 ml polypropylene tubes in a dry place. Prepare the suspension in 50 ml aliquots with 500 mg of Tetramin powder and 50 ml of NFSW. These tubes can be stored at -20°C. Tetramin tend to sediment very rapidly. When dispensing 3 ml with a plastic pipette, it is necessary to pipette up and down a few times to resuspend the powder.

Sera micron (10g/l): This is a fine powder that simply needs to be suspended in 50 ml polypropylene tubes. Use 500 mg of powder for 50 ml of NFSW. These tubes can be stored at -20°C. Sera micron tend to sediment rapidly. When dispensing 3 ml with a plastic pipette, it is necessary to pipette up and down a few times to resuspend the powder.

*Tetraselmis marina* microalgae (18 million cells/ml): This product is sold as a frozen preparation in a 1-liter bottle at a concentration of 720 million cells/ml. Before aliquoting into 50 ml polypropylene tubes, it needs to be thawed in a water bath at 37°C. These tubes are then stored at -20°C. To prepare a batch of tubes at the desired concentration, thaw a 50 ml tube and mix it with 1.95 litres of NFSW for a 40x dilution. Ensure thorough mixing and aliquot into 50 ml polypropylene tubes. Once again, these tubes are stored at -20°C.

**Fabrication of a sieve** (80 mm nylon mesh):

The sieve can be easily manufactured by using a 50 ml polypropylene tube. Using a fine saw, remove the conical bottom of the tube. Perforate the screwable lid with a cutter. Insert a piece of 80 μm nylon mesh between the tube and the lid.

**Preparation of the 7.5 % MgCl2 stock solution**

75 g of MgCl_2_ . 6 H_2_0 powder is dissolved into 900 ml of sterile deionized water. Once dissolved, complement to 1 litre with deionized water. Filter with a Millipore 0.22 μm unit. Keep at room temperature.
